# Disruption of Multiple Distinctive Neural Networks Associated With Impulse Control Disorder in Parkinson's Disease

**DOI:** 10.3389/fnhum.2018.00462

**Published:** 2018-11-21

**Authors:** Pavel Filip, Pavla Linhartová, Pavlína Hlavatá, Rastislav Šumec, Marek Baláž, Martin Bareš, Tomáš Kašpárek

**Affiliations:** ^1^First Department of Neurology, Faculty of Medicine, Masaryk University and University Hospital of St. Anne, Brno, Czechia; ^2^Center for Magnetic Resonance Research (CMRR), University of Minnesota, Minneapolis, MN, United States; ^3^Department of Psychiatry, Faculty of Medicine, Masaryk University and University Hospital Brno, Brno, Czechia; ^4^Department of Neurology, School of Medicine, University of Minnesota, Minneapolis, MN, United States

**Keywords:** impulse control disorder, Parkinson's disease, fMRI, functional connectivity, Go/No Go task, delay discounting task

## Abstract

The phenomenon of impulsivity in Parkinson's disease appears as an arduous side effect of dopaminergic therapy with potentially detrimental consequences for the life of the patients. Although conceptualized as a result of non-physiologic chronic dopaminergic stimulation, recent advances speculate on combined disruption of other networks as well. In the search for neuroanatomical correlates of this multifaceted disturbance, this study employs two distinct, well-defined tasks of close association to motor inhibition and decision-making impulsivity, Go/No Go and Delay discounting. The fMRI and functional connectivity analysis in 21 Parkinson's disease patients, including 8 patients suffering from severe impulse control disorder, and 28 healthy controls, revealed in impulsive Parkinson's disease patients not only decreased fMRI activation in the dorsolateral prefrontal cortex and bilateral striatum, but also vast functional connectivity changes of both caudate nuclei as decreased connectivity to the superior parietal cortex and increased connectivity to the insular area, clearly beyond the commonly stated areas, which indicates that orbitofronto-striatal and mesolimbic functional disruptions are not the sole mechanisms underlying impulse control disorder in Parkinson's disease. Ergo, our results present a refinement and synthesis of gradually developing ideas about the nature of impulsive control disorder in Parkinson's disease—an umbrella term encompassing various behavioral deviations related to distinct neuronal networks and presumably neurotransmitter systems, which greatly exceed the previously envisioned dopaminergic pathways as the only culprit.

## Introduction

While considered a mere movement disorder in the times past, Parkinson's disease (PD) is now generally seen as a complex dimension of multiple motor, cognitive, and behavioral components, with neuropsychiatric affections as depression, apathy, and impulse control disorders (ICDs) being the most salient of the non-motor symptoms (Cooney and Stacy, 2016). Impulsivity, commonly defined as the lack of behavioral inhibition and/or premature decision making, entails compulsive or repetitive engagement in certain activities, closely associated with the inability to foresee or learn from negative outcomes. Specifically in PD, a diverse spectrum of maladaptive behaviors is included in ICDs such as pathological gambling, paraphilias, excessive shopping, or binge eating, with the list sometimes extended by closely related phenomena and purposeless, repetitive behaviors as punding, hoarding, and hobbyism (Weintraub et al., 2015). Given the paucity of therapeutic options and potentially devastating consequences, inter alia, financial ruin, divorce, or loss of employment, the recognition of these aberrant behaviors in routine clinical practice and the delineation of precise neurobiological correlates and causes are of paramount importance.

ICDs are thought to be triggered by the interaction of chronic dopaminergic medication, especially dopamine agonist therapy (Garcia-Ruiz et al., 2014), and pathophysiological vulnerabilities, either pre-existing before the onset of the disease, or associated directly with neurodegeneration in progressing PD (Vriend, 2018), as occurrence of ICDs in treatment-naïve PD patients is very similar to the general population (Weintraub et al., 2013). The underlying neuropathology of ICD probably involves not only the overstimulation of dopaminergic reward-related pathways, hence assigning excessive salience to incentives (Robinson and Berridge, 1993), but also the interference in D2-signaling pauses in the ventral striatum (Frank et al., 2004; Vriend, 2018), which impairs the encoding of harmful behavior, i.e., prevents negative-feedback learning, and leaves D1-receptor-facilitated positive reinforcement intact. Moreover, dopamine receptor abnormities (Steeves et al., 2009; Vriend et al., 2014) support the hypothesis of PD pathology being a direct predisposition to ICD.

The previous body of MRI research in ICD has firmly established disturbances not only in the striatal regions (Gescheidt et al., 2012), but also in the limbic cortex during tasks associated with visual sexual cues (Politis et al., 2013), probabilistic learning (Voon et al., 2010), and risk taking (Voon et al., 2011a), also suggesting the dysregulation of mesolimbic dopaminergic pathways. Nonetheless, this hypothesis was partially countered by structural MRI (Biundo et al., 2015), perfusion SPECT imaging (Cilia et al., 2011), tracers with high affinity for extrastriatal D2/D3 receptors (Buckholtz et al., 2010; Ray et al., 2012) uncovering dysfunctions beyond the sole disturbance of the mesolimbic system and striatum.

With this discrepancy in mind, the presented cross-sectional study used a multimodal approach encompassing behavioral, fMRI activation and functional connectivity analysis in two distinctive tasks reflecting various aspects of impulsivity to elucidate the neurobiology underlying ICD in PD further—specifically motor response inhibition in a Go/No Go (GNG) task and decision-making impulsivity in a Delay Discounting (DD) task. Moreover, only PD patients with truly detrimental effects of ICD were selected to avoid borderline effects for activities, which may be considered not genuinely abnormal or deviant from premorbid behavior. Our premise anticipated not only affections in the striatum and mesolimbic system, but also the recruitment and connectivity changes from the striatum to other cortical areas beyond the dopamine regulated network in both tasks. Furthermore, we intended to evaluate eventual overlap of neuroanatomical signatures of impulsivity-eliciting stimuli in ICD-PD patients in both tasks to delineate the truly common nodes for distinctive, albeit impulse-control-oriented activity types.

## Methods

### Subjects

A total of twenty-eight PD patients were recruited at the 1st Department of Neurology, University Hospital of St. Anne, Brno, Czech Republic, based on the UK Brain Bank Criteria (Hughes et al., 1992). This cohort included specifically selected ten PD patients with significant signs of ICD affecting their day-to-day lives. Demographic (gender, age) and neurologic data [Hoehn & Yahr stage (Hoehn and Yahr, 1967), age at the onset of the disease, disease duration, L-dopa equivalent dose (Tomlinson et al., 2010)] were recorded, complemented with depression and impulsivity evaluation [Montgomery-Asberg Depression Scale (MADRS) (Montgomery and Asberg, 1979) and Barratt scale (Patton et al., 1995), respectively; see the Table 1]. All the assessments of PD patients, including the fMRI acquisition, were performed on medication. Additionally, we recruited twenty-nine healthy controls, who underwent the same MRI, and neuropsychological protocols as PD patients.

**Table 1:** Demographics, neurologic, neuropsychologic and behavioral data of PD subgroups and healthy controls.

	**Non-impulsive PD (*n* = 13)**	**ICD-PD (*n* = 8)**	**Healthy controls (*n* = 28)**
Gender (M/F)	5/8	6/2	14/14
Age (years)	71.0 [4.0]	65.0 [5.7]	66.4 [6.9]
**NEUROLOGIC DATA**			
H&Y stage	2.23 [0.60]	2.25 [0.53]	–
Age at the onset	65.39 [5.44]	55.25 [6.20]	–
Disease duration	5.62 [3.64]	9.75 [3.99]	–
L-dopa equivalent dose	926.67 [209.38]	1061.88 [270.70]	–
**NEUROPSYCHOLOGIC DATA**			
MADRS	3.31 [4.09]	1.63 [3.11]	0.28 [0.76]
Barratt score	53.54 [4.98]	60.88 [8.89]	55.00 [6.10]
**GNG TASK–SUCCESS RATES**			
Green cross-Go	0.89 [0.27]	0.96 [0.02]	0.93 [0.11]
Red cross-Go	0.99 [0.03]	0.89 [0.24]	0.95 [0.10]
Red cross-No Go	0.94 [0.04]	0.89 [0.11]	0.95 [0.05]
**GNG TASK – REACTION TIMES[s]**			
Green cross - Go	0.39 [0.15]	0.41 [0.09]	0.42 [0.11]
Red cross - Go	0.50 [0.07]	0.45 [0.16]	0.48 [0.09]
**DD TASK**			
Control success rate	0.95 [0.03]	0.85 [0.17]	0.97 [0.02]
Easy - immediate vs. delayed response ratio	0.44 [0.22]	0.43 [0.26]	0.47 [0.25]
Hard - immediate vs. delayed response ratio	0.41 [0.37]	0.37 [0.35]	0.48 [0.36]

*The values are stated in the format average [standard deviation]. DD, Delay Discounting task; F, female; GNG, Go/No Go task; H&Y, Hoehn & Yahr; ICD, impulse control disorder; M, male; PD, Parkinson's disease*.

We did not include individuals with conspicuous cognitive impairment [defined as Mini-mental state examination score of <27 (Folstein et al., 1975)], comorbid psychotic, affective or autistic spectrum disorder, and MRI contraindications. Furthermore, subjects with evidence of significant vascular or space occupying lesions in MRI scans and head motion beyond 3.0 mm during fMRI acquisition were excluded as well, leaving 13 non-impulsive PD patients, 8 ICD-PD patients, and 28 healthy controls.

The study was approved by the Institutional Review Board of the University Hospital of St. Anne, Brno, Czech Republic. A written informed consent was provided by each subject in accordance with the Declaration of Helsinki.

### Tasks

Before entering the MRI system, the subjects underwent a training session in both tasks to avoid misunderstanding of the instructions and the interference of eventual learning effects in the fMRI results. The subjects responded to stimuli by pressing a button with the dominant hand.

#### Go/no go

The task began with either a red or a green fixation cross displayed for the period of 2–6 s. The subjects were notified in advance that the green fixation cross (1/3 of runs) would always be followed by the Go stimulus, thus removing the need for alertness in this case. The runs with the red fixation cross (2/3 of cases) could be followed by either the Go stimulus (letter “A” displayed in the middle of the screen, presented in 1/3 of all the cases) or the No Go stimulus (letter “B” displayed in the middle of the screen, presented also in 1/3 of all the cases). The stimulus duration was 0.2 s, succeeded by a 2 s empty screen. The subject was supposed to press or avoid pressing a key based on the stimulus type. The whole task consisted of 4 blocks, each with 54 stimuli [18 Green cross–Go (GcG), 18 Red cross–Go (RcG), 18 Red cross–No Go (RcNG) runs]. The blocks were divided by short breaks.

#### Delay discounting

During this task, the subject was shown two options—an immediate and a delayed reward, with random arrangement at the left or the right side. The task included 3 types of questions: (I) difficult questions, with rewards of similar subjective value as determined in the pre-acquisition training part utilizing a well-documented and widely accepted approach (Mazur, 1987); (II) easy questions with options of distinctive subjective value, and (III) control questions, where one of the responses was associated with significant objective advantage over the other (4 types: naught now vs. some reward later; some reward now vs. naught later; the same reward now and later; higher reward now than later). The options were shown for 7 s, followed by a 1-second-long blank screen. The subject was required to press a key corresponding to the chosen option, highlighting the desired response. The whole task consisted of three blocks, each with 48 stimuli (16 questions of each type). The blocks were divided by short breaks.

### MRI data acquisition

MRI scanning was performed using a 3 Tesla whole body MRI scanner SIEMENS MAGNETOM Prisma syngo (Siemens Medical Systems, Erlangen, Germany) at the Central European Institute of Technology, Brno, Czech Republic. At the beginning, a high-resolution anatomical T1-weighted scan was acquired with the following parameters: magnetization-prepared rapid gradient-echo (MPRAGE) sequence [repetition time (TR) = 2,300 ms, echo time (TE) = 2.34 ms, flip angle (FA) = 8°, voxel size 1.00 × 1.00 × 1.00 mm, slice thickness 1.00 mm, matrix 240 × 224 × 224]. Subsequently, whole brain fMRI was performed with the parameters: TR = 2280 ms, TE = 35.0 ms, FA = 75°, voxel size 3 × 3 × 3 mm, 39 sagittal slices, field of view 192 × 192 mm, total number of volumes 153 per one block of the GNG task (i.e., 612 volumes in total) and 175 per one block of the DD task (i.e., 525 volumes in total).

### Analysis of demographic and behavioral data

Firstly, equivalence analysis [two-one-sided test (Schuirmann, 1987)] was used to confirm the absence of significant differences in basic demographic parameters between PD patients and healthy controls (gender, age). Furthermore, where we expected the subgroups to differ, analysis of variance (ANOVA) was used to evaluate the parameters in the individual groups (MADRS, Barratt score).

In the behavioral analysis of the performance in the two tasks, the primary parameters of interest in the GNG task included the success rate and reaction times in individual stimuli variants and in the DD task, the percentage of correct responses in the control trials, and immediate vs. delayed responses for easy and hard trials. The average success rates and reaction/response times were determined for each subject in order to use parametric statistical analyses. ANOVA was used to compare the individual subject groups—non-impulsive PD, ICD-PD and healthy controls. All the analyses were performed using Statistica 13 (Statsoft Inc., Oklahoma, USA).

### Analysis of fMRI data

MRI data were processed and analyzed using SPM12 (Wellcome Department of Cognitive Neurology, London, UK) implemented in Matlab R2017b (Mathworks Inc., Natick, MA, USA). The preprocessing of fMRI images included the realignment to correct for the movements of the subject's head. As stated in the section Subjects, the threshold of 3 mm shift and 3° rotation in any direction was implemented, excluding 8 subjects in total. Subsequently, co-registration of functional and anatomical images and interpolation in time were performed, followed by the spatial normalization into the stereotactic Montreal Neurological Institute (MNI) space and spatial smoothing (isotropic Gaussian kernel of 8 mm full-width at half-maximum). The data were high-pass filtered with a Gaussian kernel filter of 128 s.

The first level general linear model of BOLD activations in the GNG task included the time windows between the stimulus presentation (letter “A” or “B” distinguishing the Go and No Go runs) and key press by the subject. The individual design matrix for each subject distinguished the GcG runs, RcG runs, and RcNG runs, including the accuracy of the response (key pressed correctly in the Go task and key not pressed in the No Go task) and the head movements in all the directions as nuisance covariates, thus providing three contrast maps. These were then submitted to the second level full factorial design (3 × 3) with the following factors: subject subgroups (non-impulsive PD, ICD-PD, healthy controls) and 3 run types (GcG, RcG, RcNG), and the age and gender as covariates of non-interest.

In the DD task, the first level general linear model consisted of the time windows between the stimulus presentation (2 windows with 2 options to choose from) and the subject's key press. The design matrix of individual subjects included the control, easy and hard task types, with the head movements as nuisance covariates. Once again, the three generated contrast maps were submitted to the second level full factorial design (3 × 3) analysis with the following factors: subject subgroups (non-impulsive PD, ICD-PD, healthy controls) and 3 run types (control, easy and hard), and the age and gender as covariates of non-interest.

Furthermore, we analyzed the differences in the individual sub-groups in the task-related connectivity. The analysis of psychophysiological interactions [PPI (Friston et al., 1997)] focused on the connectivity of striatum bilaterally, specifically the caput of the caudate nuclei, as defined by the Automated Anatomical Labeling (AAL) atlas (Tzourio-Mazoyer et al., 2002; Maldjian et al., 2003), repeatedly emerging in the previous activation analysis. The time course of these two individual seeds (the caput of the left and the right caudate nucleus) was extracted as the average over the atlas-defined volume of interest in both tasks. The two created first level models consisted of the extracted series, the run type regressor (GcG, RcG, and RcNG in the GNG task and control, easy and hard in the DD task), the PPI regressor, and the head movements in all the directions as nuisance covariates, thus providing three individual t-contrasts. These were then submitted to the two subsequent second level analyses with the full factorial designs corresponding to the constructs in the activation analysis, i.e., subject groups (non-impulsive PD, ICD-PD, healthy controls) and 3 run types (GcG, RcG, RcNG, and control, easy and hard in the GNG and DD tasks, respectively). Furthermore, age and gender were included as nuisance variables.

### Statistical thresholds

Due to the relative paucity of data points, the behavioral and demographic results are presented at the significance level of *p* < 0.05. Secondly, the activation analysis results were considered significant at *p* < 0.05, family-wise error (FWE)-corrected for multiple comparisons at the voxel level (with the cluster threshold of 40 contiguous voxels). And because of the nature and utilized contrasts, the PPI analysis adopted a less stringent threshold of *p* < 0.05, FWE-corrected for multiple comparisons at the cluster level (voxel-wise threshold of *p* < 0.001, uncorrected, small volume correction, cluster threshold of 40 contiguous voxels).

## Results

### Characteristics of the subjects and behavioral performance

The two-one-sided test showed no difference in age (*p* = 0.045) and gender (*p* = 0.035) between the PD patients and controls (with 5-year and 25% mean difference considered clinically relevant for the age and the gender, respectively). However, implementing the same hypothesized mean differences, the subgroups of ICD-PD patients and non-impulsive PD patients cannot be considered equivalent (*p* > 0.2 for both age and gender). As for the clinical data, the ICD-PD and non-impulsive PD groups had similar modified Hoehn & Yahr stage (*p* < 0.001) and L-dopa equivalent dose (*p* = 0.37; with 1 stage and 300 mg L-dopa equivalent dose mean difference considered clinically relevant). Nonetheless, there was a significant difference in both the age at the PD onset and disease duration (*p* > 0.20 for both comparisons, with 5-year and 2-year difference considered clinically relevant, respectively).

Moreover, ANOVA revealed significant differences among the subgroups in both MADRS [*F*_(2, 47)_ = 6.64, *p* = 0.003] and Barratt scale [*F*_(2, 47)_ = 3.57, *p* = 0.036], with the highest depression scores found in the non-impulsive PD group, and, the highest impulsivity scores, as expected, in the ICD-PD group.

ANOVA in the GNG task found no significant between-group differences in the success rates [*F*_(2, 47)_ = 0.46, 1.42 and 2.06 in the GcG, RcG, and RcNG tasks, respectively, with p >0.10 for all the comparisons) and reaction times [*F*_(2, 47)_ = 0.24 and 0.62 for GcG and RcG tasks, respectively, with *p* >0.50 for both comparisons]. No group differences were revealed also in the analysis of the immediate vs. delayed response ratios in the easy and hard stimuli types in the DD task [*F*_(2, 47)_ = 0.12 and 0.39 for easy and hard, respectively, with *p* >0.50 for both comparisons]. Nonetheless, a significant distinction was found in the ratio of correct responses to the control task [*F*_(2, 47)_ = 8.10, *p* = 0.001], with lower success rate in the ICD-PD patients. For more information, see the Table 1.

### Activation and connectivity analysis

Due to the sheer extent of acquired results, only data relevant for ICD-PD patients are reported to avoid the dilution of consequential outcomes.

**Figure 1:**
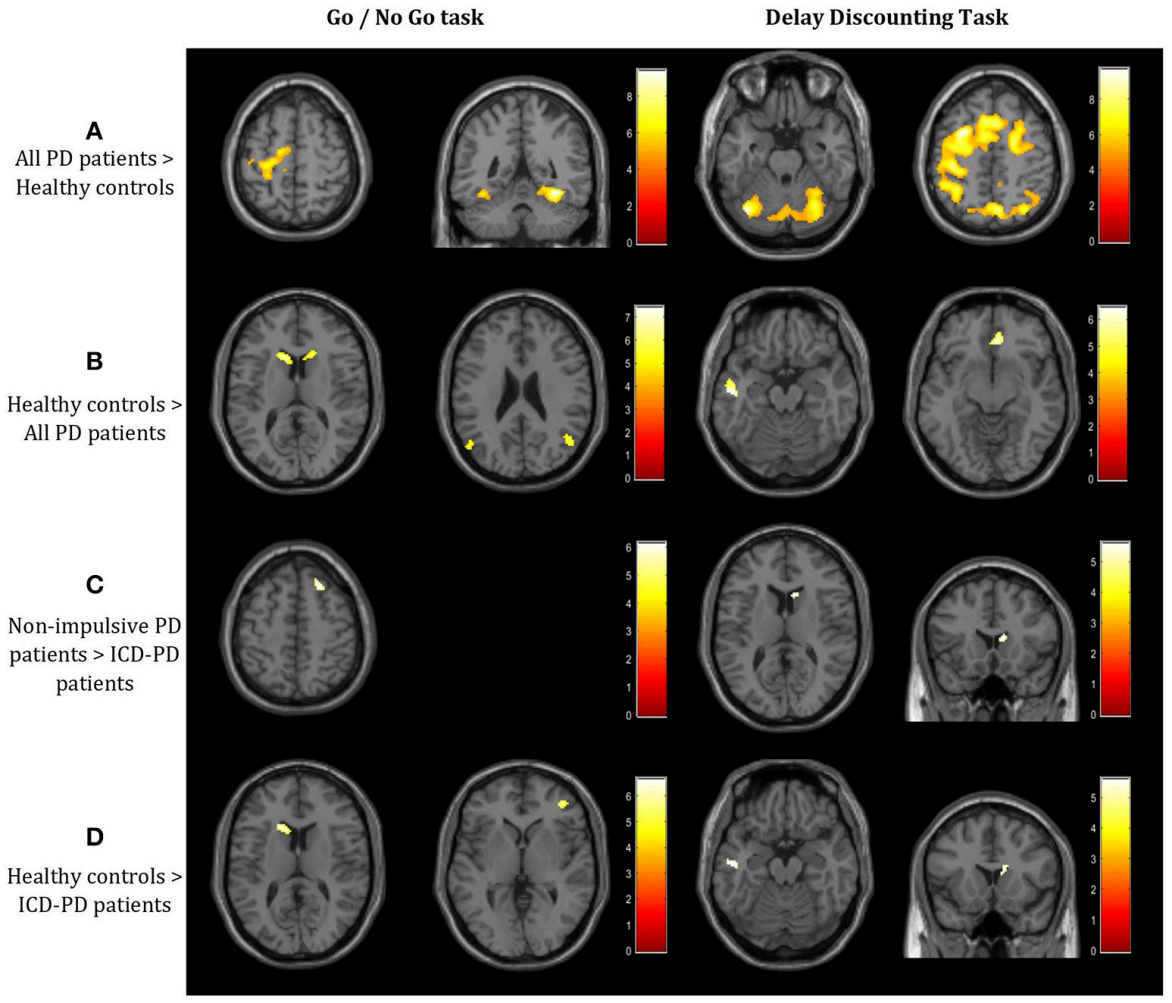
Results of the 3 × 3 full factorial design (3 subgroups vs. 3 stimuli types) in 2 tasks – Go/No Go (GNG) and Delay Discounting (DD; *p* < 0.05, FWE-corrected at the voxel level; T-contrasts, threshold T = 4.73 for both GNG and DD tasks). (**A**) Increased activation in the right precentral, both fusiform gyri, cerebellum, and vast precentral and postcentral areas, including the dorsolateral prefrontal cortex in the whole cohort of Parkinson's disease (PD) patients when compared to healthy controls. (**B**) Decreased activation in bilateral caudate, angular gyri, left middle temporal and right cingulate gyrus. **(C)** Decreased activation in the superior frontal gyrus and right striatum in ICD-PD patients when compared to non-impulsive PD patients. (**D**) Decreased activation in both left and right caudate and the right dorsolateral prefrontal cortex in PD patients with impulsivity when compared to healthy controls. Laterality conventions with the right side in the figure corresponding to the right side of the scanned area were implemented. See Table 2 for detailed statistical results and anatomical localization of clusters.

Between-group contrasts revealed the following differences in the combined outcome of all stimuli types (not distinguishing between the Go and No Go runs in the GNG task, and control, easy and hard stimuli in the DD task):

- **All PD patients** > **Healthy controls**—As illustrated in the Figure 1A and detailed in the Table 1, PD patients had significantly higher activation in various cortical areas in both tasks, including the left supplementary motor cortex, bilateral fusiform gyrus (GNG task), and vast cortical areas around the left central sulcus, in both thalami and both lobuli VI of the cerebellum (DD task)
- **Healthy controls** > **All PD patients—**PD patients showed lower activity in both caudate nuclei and angular gyri (GNG task) and left middle temporal gyrus and anterior cingulate (DD task; see Figure 1B, Table 1).
- **Non-impulsive PD patients** > **ICD-PD patients—**Right Brodmann area 8 (GNG task) and right caudate (DD task) were less active in ICD-PD patients (see Figure 1C, Table 1). The effects of the reverse contrast failed to reach the predetermined significance thresholds.
- **Healthy controls** > **ICD-PD patients—**The hypoactivity of caudate nuclei was also implicated in this contrast in PD-ICD patients (left-side in the GNG task, right side in the DD task). Moreover, right dorsolateral prefrontal cortex (DLPFC) and left middle temporal lobe were less active in PD-ICD patients (see Figure 1D, Table 1). Again, effects of the reverse contrast were not significant.

**Table 2:** Anatomical localization of clusters in the activation analysis in the Go/No Go and Delay Discounting tasks.

	**Anatomical regions**	**Brodmann area**	**Side**	**Volume (in voxels)**	**p-value (SVC)**	**T-score of local max**	**MNI coordinates of local maxima**		
**Go/No go task**	**ALL PD PATIENTS** > **HEALTHY CONTROLS**								
	Fusiform gyrus	BA 37	R	434	< 0.001	9.42	34	−48	−14
	SMA	BA 6	L	927	< 0.001	7.16	−8	−14	48
	Precentral gyrus	BA 6	L	117	< 0.001	6.44	−54	0	42
	Fusiform gyrus	BA 37	L	103	< 0.001	6.40	−38	−50	−16
	SMA, middle cingulate	BA 32, 6	L	141	< 0.001	6.36	−12	10	42
	SMA	BA 6	R	86	< 0.001	6.18	12	16	44
	Postcentral gyrus		L	143	< 0.001	6.10	−34	−20	40
	Calcarine sulcus		L	52	0.002	5.62	−10	−68	10
	Supramarginal gyrus	BA 40	L	58	0.002	5.57	−48	−40	32
	Calcarine sulcus		R	75	0.002	5.56	14	−72	12
	Cuneus		R	73	0.002	5.51	−10	−88	28
	Precentral gyrus	BA 6	R	55	0.003	5.47	36	4	46
	**HEALTHY CONTROLS** > **ALL PD PATIENTS**								
	Inferior occipital lobe	BA 18	L	76	< 0.001	7.45	−28	−90	−6
	Inferior occipital lobe	BA 18	R	82	< 0.001	7.03	34	−84	−6
	Caudate		L	97	< 0.001	6.82	−12	20	14
	Caudate		R	43	< 0.001	6.18	18	24	14
	Angular gyrus	BA 39	R	50	0.002	5.60	48	−60	24
	Angular gyrus	BA 39	L	46	0.002	5.43	−50	−70	26
	**NON-IMPULSIVE PD PATIENTS** > **ICD-PD PATIENTS**								
	Frontal superior gyrus	BA 8	R	60	< 0.001	6.16	22	28	56
	**HEALTHY CONTROLS** > **ICD-PD PATIENTS**								
	Superior frontal gyrus	BA 8	R	70	< 0.001	6.68	22	28	54
	Caudate		L	62	< 0.001	6.38	−14	20	14
	DLPFC	BA 10	R	48	0.001	5.67	40	46	4
**Delay Discounting Task**	**ALL PD PATIENTS** > **HEALTHY CONTROLS**								
	Precentral gyrus, SMA	BA 6	L	17,823	< 0.001	9.72	−26	8	50
	Thalamus Thalamus		L R	1,756	< 0.001	9.08	−10 14	−18 −16	4 2
	Cerebellum, lobule VI Cerebellum, lobule VI		R L	2,361	< 0.001	8.85	24 −36	−92 −68	−4 −24
	Cingulate gyrus	BA 31	R	152	< 0.001	6.64	10	−40	46
	Supramarginal gyrus	BA 40	L	52	0.001	5.76	−54	−44	24
	Cerebellum, vermis III		R	65	0.001	5.75	2	−44	−18
	Dorsolateral prefrontal cortex	BA 46	R	54	0.002	5.60	50	46	6
	**HEALTHY CONTROLS** > **ALL PD PATIENTS**								
	Middle temporal gyrus	BA 21	L	145	< 0.001	6.48	−50	−14	−20
	Dorsal anterior cingulate area	BA 32	R	40	< 0.001	6.10	6	34	−10
	**NON-IMPULSIVE PD PATIENTS** > **ICD-PD PATIENTS**								
	Caudate		R	45	0.003	5.51	12	18	12
	**ICD-PD PATIENTS** > **HEALTHY CONTROLS**								
	Middle frontal lobe	BA 6	L	217	< 0.001	7.25	−26	8	50
	Thalamus Thalamus		L R	640	< 0.001	6.75	−8 10	−16 −26	2 −4
	Lingual gyrus	BA 30	L	789	< 0.001	6.73	−22	−70	6
	Precentral gyrus	BA 6	R	81	< 0.001	6.37	50	0	34
	Precentral gyrus	BA 6	R	216	< 0.001	6.36	22	−2	48
	Fusiform gyrus		R	85	< 0.001	6.33	36	−64	−10
	Precentral gyrus	BA 6	L	262	< 0.001	6.16	−36	−12	34
	Inferior parietal lobe	BA 40	L	154	0.001	5.69	−28	−48	32
	Lingual gyrus	BA 30	L	63	0.008	5.22	−16	−52	2
	**HEALTHY CONTROLS** > **ICD-PD PATIENTS**								
	Caudate		R	59	0.002	5.63	14	14	20
	Middle temporal lobe	BA 21	L	50	0.002	5.60	−50	−14	−18

*Clusters significant at p < 0.05, FWE-corrected for multiple comparisons at the voxel level (with the cluster threshold of 40 contiguous voxels). Abbreviations: BA, Brodmann area; DD, Delay Discounting task; DLPFC, dorsolateral prefrontal cortex; few, family-wise error; GNG, Go/No Go task; ICD, Impulse control disorder; L, left; MNI, Montreal Neurological Institute; PD, Parkinson's disease; R, right; SMA, supplementary motor area*.

Further contrasts of PD-ICD patients, including interaction analyses with the task types, failed to reveal any significant clusters at the predetermined threshold.

The seeds for the PPI analysis were localized in the striatum, with more precise focus on both the heads of nuclei caudate based on the activation analysis results above. As the more relevant contrasts using stimuli supposedly associated with higher impulse control requirements provided far more significant and pertinent results, only these outcomes (i.e., RcNG stimulus and the difference of RcNG > RcG in the GNG task, and Easy choice (EC) and the difference of Easy choice > Control stimuli (EC > CS) in the DD task) are reported.

**Figure 2:**
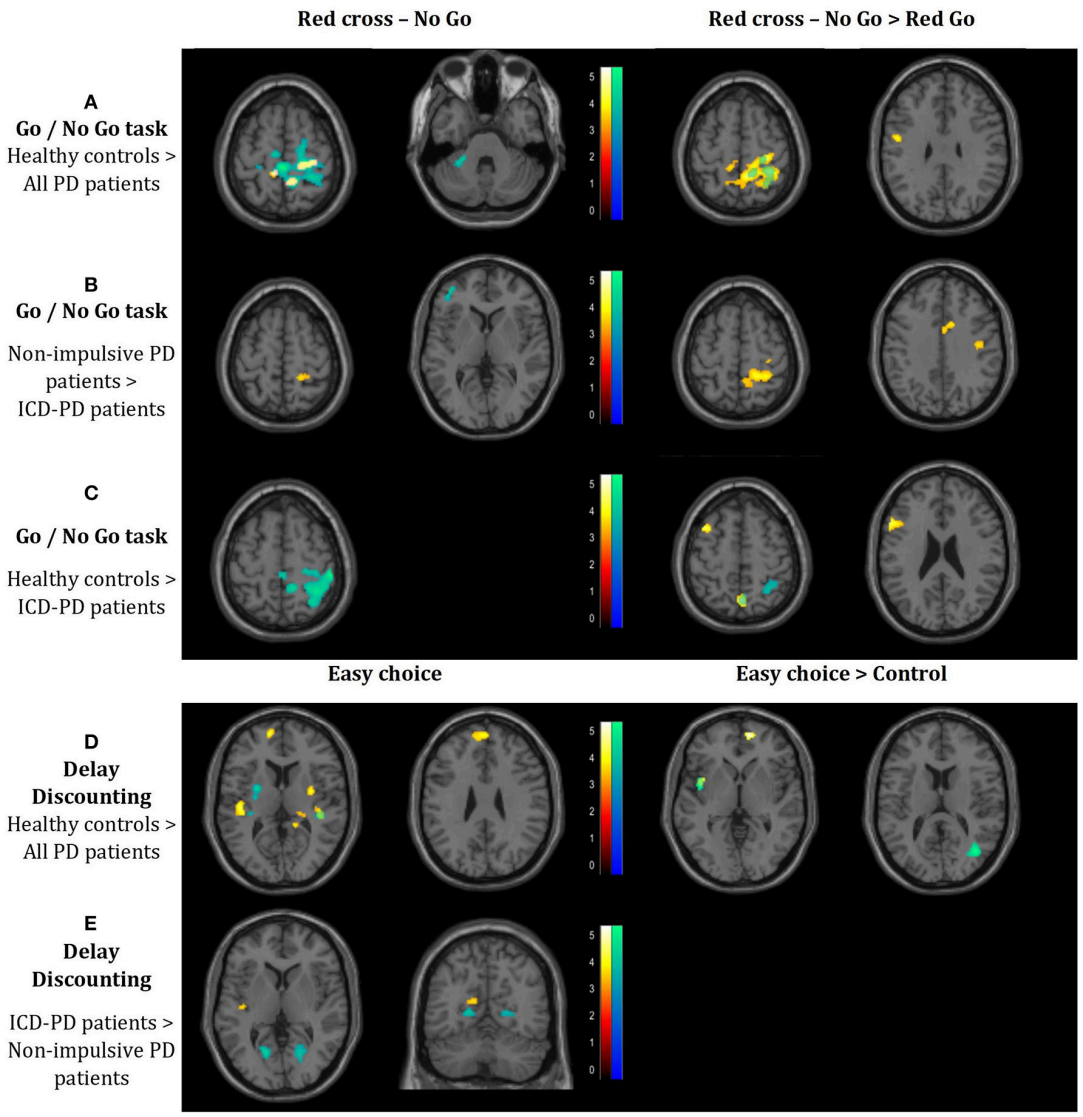
Results of the connectivity analysis (*p* < 0.05) FWE-corrected at the cluster level (p < 0.001 uncorrected at the voxel level; threshold T = 3.16 for all the reported results). The results of the functional connectivity of the seed in the left caudate head and in the right caudate head are denoted by red-to-yellow and blue-to-green spectrum, respectively. Results of the connectivity analysis in the Go/No Go task: **(A)** Decreased connectivity in the whole cohort of Parkinson's disease (PD) patients to the right-side postcentral cortical areas, left precentral gyrus and left cerebellar lobule VI, when compared with healthy controls. **(B)** Decreased connectivity in PD patients with impulse control disorder (ICD) vs. non-impulsive PD patients to the left dorsolateral prefrontal cortex, cingulate gyrus and right-side postcentral cortical areas. **(C)** Decreased connectivity in ICD-PD patients vs. healthy controls to the right-side postcentral cortical areas and left dorsolateral prefrontal cortex. Results of the connectivity analysis in the Delay Discounting task. **(D)** Decreased connectivity in the whole cohort of PD patients to both putamina (each contralateral to the respective seed), both superior temporal gyri (each ipsilateral to the respective seed) and left medial frontal cortex, when compared with healthy controls. **(E)** Increased connectivity in ICD-PD patients vs. non-impulsive PD patients to the left insula and bilateral calcarine cortices. Laterality conventions with the right side in the figure corresponding to the right side of the scanned area were implemented. See Table 3 for detailed statistical results and anatomical localization of clusters.

- **GNG task–Healthy controls** > **All PD patients:** This comparison revealed decreased connectivity in PD patients dominantly to the right-side postcentral cortical areas from both the right and the left seed in both the contrasts (simple RcNG, and RcNG > RcG), furthermore to the left precentral gyrus (from the left caudate in the RcNG > RcG contrast) and the left cerebellar lobule VI (from the right caudate in the RcNG contrast), when compared with healthy controls (see Figure 2A, Table 3).
- **GNG task–Non-impulsive PD patients** > **ICD-PD patients:** This analysis yielded decreased connectivity in ICD-PD patients to the left DLPFC (from the contralateral caudate) and decreased connectivity from the left caudate to the right superior parietal cortex in both the used contrasts and to the right cingulate gyrus in the RcNG >RcG contrast (see Figure 2B, Table 3).
- **GNG task–Healthy controls** > **ICD-PD patients:** ICD-PD patients showed decreased connectivity of the right caudate to the right superior parietal cortex in both contrasts and decreased connectivity of the left caudate to the ipsilateral DLPFC (see Figure 2C, Table 3).
- **DD task–Healthy controls** > **All PD patients:** Decreased connectivity of both caudate nuclei to the contralateral putamina was found in the pooled PD patient group in the simple EC contrast. Furthermore, PD patients had decreased connectivity of caudate nuclei to ipsilateral superior temporal gyri and both the left (simple EC contrast) and the right (EC > CS contrast) medial frontal cortex from the left caudate (see Figure 2D, Table 3).
- **DD task–ICD-PD patients** > **Non-impulsive PD patients:** The simple EC contrast revealed increased connectivity of the right caudate to bilateral calcarine cortices and increased connectivity of the left caudate to the ipsilateral insula in ICD-PD patients (see Figure 2E, Table 3).

**Table 3:** Anatomical localization of clusters in the connectivity analysis in the Go/No Go and Delay Discounting tasks.

	**Anatomical regions**	**Brodmann area**	**Side**	**Volume (in voxels)**	***p*-value (SVC)**	**T-score of local max**	**MNI coordinates of local maxima**		
**Go/No go task**	**RED CROSS NO GO IN HEALTHY CONTROLS** > **ALL PD PATIENTS**								
	**Seed in the left caudate head**								
	Precentral gyrus	BA 4	R	154	0.002	4.02	26	−28	64
	Precuneus	BA 5	R	106	0.003	3.84	8	−46	62
	Precuneus		L	44	0.003	3.70	−8	−36	62
	**Seed in the right caudate head**								
	Postcentral gyrus Precentral gyrus	BA 6, 40, 4	R	2,055	0.001	5.76	22	−28	66
	Paracentral lobule	BA 6	L	77	0.002	4.38	−8	−18	64
	Cerebellum, lobule VI		L	55	0.007	4.06	−34	−46	−34
	Postcentral gyrus	BA 3, 4	L	57	0.003	3.94	−20	−32	68
	**RED CROSS NO GO IN NON-IMPULSIVE PD PATIENTS** > **ICD-PD PATIENTS**								
	**Seed in the left caudate head**								
	Postcentral gyrus	BA 3	R	55	0.003	3.66	20	−40	60
	**Seed in the right caudate head**								
	DLPFC	BA 10	L	62	0.004	3.94	−38	50	4
	**RED CROSS NO GO IN HEALTHY CONTROLS** > **ICD-PD PATIENTS**								
	**Seed in the right caudate head**								
	Postcentral gyrus	BA 40, 7	R	1,223	0.001	4.66	22	−28	66
	Precuneus	BA 5	R	254	0.001	4.44	10	−46	62
	Precuneus	BA 7	R	61	0.003	3.95	8	−60	52
	Postcentral gyrus	BA 4	L	45	0.004	3.66	−22	−30	66
	**RED CROSS NO GO** > **RED CROSS GO IN HEALTHY CONTROLS** > **ALL PD PATIENTS**								
	**Seed in the left caudate head**								
	Postcentral, precuneus	BA 5, 7	R	1,266	< 0.001	4.53	28	−26	62
	Superior parietal lobe	BA 7	L	67	0.003	4.41	−20	−80	46
	Precentral gyrus	BA 6	L	82	0.002	3.90	−10	−36	64
	Middle temporal lobe	BA 22	L	70	0.003	3.79	−50	−4	30
	**Seed in the right caudate head**								
	Postcentral gyrus	BA 40, 7	R	246	0.001	4.11	32	−40	60
	Postcentral gyrus	BA 4	R	129	0.002	4.04	26	−28	62
	Postcentral gyrus	BA 5	R	60	0.002	3.89	14	−42	60
	**RED CROSS NO GO** > **RED CROSS GO IN NON-IMPULSIVE PD PATIENTS** > **ICD-PD PATIENTS**								
	**Seed in the left caudate head**								
	Caudate		R	47	0.003	4.18	16	−20	26
	Postcentral gyrus	BA 5	R	441	0.001	3.97	28	−40	64
	Cingulate gyrus	BA 24	R	61	0.004	3.73	12	10	36
	Precentral gyrus	BA 6	R	58	0.003	3.69	44	−14	34
	**RED CROSS NO GO** > **RED CROSS GO IN HEALTHY CONTROLS** > **ICD-PD PATIENTS**								
	**Seed in the left caudate head**								
	Precuneus	BA 7	C	106	0.001	4.60	2	−64	56
	Superior parietal lobe	BA 7	L	73	0.004	4.50	−20	−78	48
	Angular gyrus	BA 40	L	55	0.004	4.44	−46	−66	46
	DLPFC	BA 9	L	285	0.002	4.23	−54	20	26
	Middle frontal gyrus	BA 6	L	74	0.004	4.22	−40	16	54
	**Seed in the right caudate head**								
	Superior parietal gyrus	BA 40	R	129	0.002	3.89	30	−54	58
	Precuneus	BA 7	R	47	0.003	3.84	4	−64	56
**Delay discounting task**	**EASY STIMULUS IN HEALTHY CONTROLS** > **ALL PD PATIENTS**								
	**Seed in the left caudate head**								
	Thalamus		R	395	0.001	4.57	20	−28	14
	Superior temporal gyrus	BA 41	L	117	0.002	4.48	−44	−24	12
	Superior temporal gyrus	BA 41	R	162	0.001	4.19	44	−30	14
	Medial frontal cortex	BA 10	L	42	0.004	4.12	−12	58	10
	Putamen		R	43	0.004	3.92	32	−4	10
	Medial frontal gyrus	BA 9, 10	C	97	0.003	3.89	0	56	28
	**Seed in the right caudate head**								
	Putamen		L	137	0.003	4.06	−28	−24	6
	Superior temporal gyrus	BA 41	R	90	0.002	3.93	42	−32	10
	**EASY STIMULUS IN ICD-PD PATIENTS** > **NON-IMPULSIVE PD PATIENTS**								
	**Seed in the left caudate head**								
	Insula	BA 13	L	47	0.003	3.74	−42	−18	8
	Calcarine		L	44	0.004	3.61	−12	−60	16
	**Seed in the right caudate head**								
	Calcarine	BA 30	L	91	0.002	4.20	−16	−66	4
	Calcarine	BA 30	R	95	0.002	3.85	20	−66	4
	**EASY STIMULUS** > **CONTROL STIMULUS IN HEALTHY CONTROLS** > **ALL PD PATIENTS**								
	**Seed in the left caudate head**								
	Medial frontal cortex	BA 10	R	59	0.003	4.07	12	56	0
	Insula	BA 13	L	41	0.004	3.41	−40	8	2
	**Seed in the right caudate head**								
	Middle temporal gyrus		R	128	0.001	4.01	38	−68	14
	Insula	BA 13	L	70	0.002	3.83	−42	6	2

*Clusters significant at p < 0.05, FWE-corrected for multiple comparisons at the cluster level (voxel-wise threshold of p < 0.001, uncorrected, small volume correction). BA, Brodmann area; C, central; DD, Delay Discounting task; DLPFC, dorsolateral prefrontal cortex; few, family-wise error; GNG, Go/No Go task; ICD, Impulse control disorder; L, left; MNI, Montreal Neurological Institute; PD, Parkinson's disease; R, right; SVC, small volume correction*.

No clusters survived the reverse contrasts to the above stated outcomes at the same threshold.

## Discussion

This study is the first to investigate the neural substrates of ICD in PD using two distinct, impulse-control-related fMRI tasks in the same patient population, with a specific focus on cases of severe ICD significantly impacting the quality of life. Neuroimaging analyses revealed not only decreased fMRI activation in the striatum in ICD-PD patients in keeping with the previous research reports (Napier et al., 2015; Vriend, 2018), but also vast connectivity changes beyond the commonly stated areas, indicating that fronto-striatal and mesolimbic functional disruptions are not the sole mechanisms underlying ICD in PD patients.

While ICD-PD patients did not perform differently from healthy controls and non-impulsive PD patients in the behavioral aspect of GNG and DD tasks, the inclusion of both decision-making impulsivity as a measure of mapping future actions into rewards in PD (Averbeck et al., 2013), and motor response inhibition tests (Nombela et al., 2014) proved of paramount importance in the fMRI analysis, with distinct patterns of concurrence in the cortical areas around the central sulcus recruited in both tasks generally less in the PD population. Moreover, the emergence of bilateral supplementary motor area hypoactivity in PD patients during the GNG task, previously proven of critical importance for the selection of appropriate responses and the inhibition of the inappropriate ones, and fronto-parietal cortices is in accord with processes implicated in motor response inhibition (Simmonds et al., 2008) and clearly shows the encroachment of neurodegeneration processes to a wide-spread network of distinctive neural nodes. Frontal and parietal areas partly share one inhibitory-attentional network associated with action withholding and interference inhibition (Levy and Wagner, 2011; Sebastian et al., 2013), with substantial involvement of the fronto-striatal pathways mainly in the inhibition of already initiated actions (Jahfari et al., 2011). The fronto-parietal recruitment changes in non-PD gamblers, supposedly reflecting the cue-induced addiction memory network (Miedl et al., 2010), may mirror processes comparable to the dysfunctions presented in this study.

Nonetheless, the abundance of relevant research findings seems clearly to show dominant association of ICD in PD with hyperdopaminergic state and relevant structures. However, the extent, to which the function of ventral striatum, one of the core nodes implicated in ICD in PD patients, is disrupted remains equivocal, despite the mounting evidence in various studies—both increased striatal fMRI activation, in pathological gambling (Frosini et al., 2010) and hyperlibidinous deviations (Politis et al., 2013), and decreased neural activity in this area in risk taking activities (Rao et al., 2010; Voon et al., 2011a) in ICD-PD patients. Connectivity studies provide further prima facie evidence, with the reports of absence of connectivity differences in ICD-PD of the ventral striatum, but of decreased connectivity of the associative striatum to various frontal cortical areas (Carriere et al., 2015), even underlined by structural analyses in general PD population (Rae et al., 2012). Moreover, despite the surmised relevance of the associative striatum to cognition, primarily executive functions (Monchi et al., 2006; O'Callaghan et al., 2014), the studies concentrating on this realm in PD-ICD provided largely negative outcomes (Djamshidian et al., 2011; Antonini et al., 2017). The decreased GNG task connectivity of both caudate nuclei to the left DLPFC and of the left caudate to the cingulate gyrus in ICD-PD patients presented in our study seems to resonate well with these findings, but the suggested overall striatal connectivity decline in ICD-PD is countered by the elevation of DD-task connectivity to the salience-associated left insular regions implicated also in addiction and several neuropsychiatric disorders (Uddin, 2015). Hence, these results support the recent gradual opinion shift from simplistic views of striatal hypoactivity and hypoconnectivity to more open, structural and functional changes in the dopaminergic system in ICD-PD, where different aspects of inhibition control stem from distinct networks (Antonelli et al., 2014; Napier et al., 2015) and impulsivity is truly taken as an umbrella term encompassing multiple different behaviors and neuronal nodes (Vriend, 2018).

Indeed, the progressing PD pathology eventually affects also other neurotransmitter systems beyond the dopaminergic network—particularly noradrenaline and serotonin producing neurons (Braak et al., 2003), with suggested distinct effects also on impulsive behaviors (Vriend, 2018). This hypothesis is supported by neuroimaging of selective serotonin reuptake inhibitor (SSRI) induced modulation of response inhibition in PD patients (Ye et al., 2014) and the behavioral effects of SSRI, albeit ranging from the reduction of impulsive actions (Homberg et al., 2007; Baarendse and Vanderschuren, 2012) to the absence of effect in other impulsivity subdomains (Bari et al., 2009; Baarendse and Vanderschuren, 2012). Moreover, the atomoxetine-induced facilitation of noradrenergic signaling has been reported to reduce decision-making impulsivity and risk taking in PD patients (Kehagia et al., 2014), similarly with hypothesized dependence on impulsive behavior subtypes (Bari et al., 2009), and opioid receptor antagonists, despite the lack of clinically relevant effect in ICD-PD patients (Papay et al., 2014), are able to reduce pathological gambling (Grant et al., 2008) and improve symptoms in the impulsive-compulsive spectrum disorders (Piquet-Pessôa and Fontenelle, 2016) in PD-unrelated impulsivity.

And likewise, different aspects of impulse control may be differentially sensitive to dopamine concentration decline and pharmacologic supplementation (Voon et al., 2011a). There is an ample and growing body of research on the escalated reward-related striatal dopaminergic activity as the primary pathophysiological basis of ICD in PD, be it dominantly due to the “overdose” theory postulating excessive dopamine stimulation of the relatively preserved ventral striatum (Voon et al., 2011b), denervation-induced D3 receptor hypersensitivity in the same area (Prieto et al., 2011) or the interference in D2-signaling pauses in the ventral striatum impairing the encoding of harmful behaviors (Frank et al., 2004; Vriend, 2018). However, as a large proportion of PD patients do not develop impulsivity problems, it is less evident whether or not this specific hypodopaminergic condition and hence the increased vulnerability to ICD is wrought by antecedent neural or genetic traits, plastic structural changes in the reward system (Biundo et al., 2015) or merely a maladaptive response to non-physiological chronic dopaminergic stimulation, thus adding to the high heterogeneity of PD (Lewis et al., 2005; Farrer, 2006; van Balkom et al., 2016).

These multifaceted aspects of impulsivity and PD in general partly hamper clear-cut interpretation of the outcomes of this study, as the high diversity of impulsivity profiles and, indeed, probable subtypes of PD itself, undoubtedly interfered with the results, but the small number of ICD-PD subjects in our sample prevented any meaningful separate analyses. The low numbers of subjects presumably also contributed to the absence of significant between-group differences in the behavioral analysis, even though the simple numerical comparison seems to show at least a trend toward lower performance and the correlates of higher impulsivity in the ICD-PD group. This was primarily incurred by our deliberate decision to include only PD patients with ICD severity of detrimental extent for their day-to-day life (e.g., substantial financial losses due to gambling). Even though the prevalence of ICD in PD patients is usually reported at the level of ~15%, with non-negligible dependence on cultural factors and gender (Perez-Lloret et al., 2012; Santangelo et al., 2013; Maloney et al., 2017), ICD of the level deliberately chosen for this study is rather rare, making the recruitment of larger cohorts of severe ICD PD patients virtually impossible. As the symptoms widely range in severity, subclinical ICD symptom screenings yield significantly higher rates (Joutsa et al., 2012; Vriend et al., 2014), but these behaviors should generally be considered a disorder only when becoming harmful to the patient or interfering with the daily functioning as a significant deviation from premorbid behavior. Interestingly, most patients and caregivers do not consider their ICD a severe problem (Garcia-Ruiz et al., 2014). Ergo, the recruitment of these “borderline” patients could induce unwelcome interference in the outcomes. Secondly, the difference in age and gender between the ICD-PD and non-impulsive PD group, even though in accord with the previous body of research on risk factors of ICD (Ceravolo et al., 2009), calls also for caution in the further interpretation of differences between these two subgroups due to possible confounding. Furthermore, physiological noise, not accounted for in our study, might have led to potential distortion of the connectivity analysis outcomes, even though the analysis itself was purely task based. And lastly, our study shares the problem of all the cross-sectional research projects comparing ICD-PD and non-impulsive PD patients, as it is virtually impossible to delineate the true cause of neurobiological differences, i.e., antecedent characteristics or true alterations associated with ICD. Nonetheless, prospective fMRI study capable of recruiting a satisfactory number of high-severity ICD-PD patients is highly impractical, if feasible at all, and the general character of our hypotheses allows reasonable confidence in the outcomes.

In conclusion, our results present a refinement and synthesis of gradually developing ideas about the nature of ICD in PD—an umbrella term encompassing various behavioral deviations related to distinct neuronal networks, which greatly exceed the previously envisioned fronto-striatal and mesolimbic pathways. The significance of these differences in the context of disruptions to neurotransmitter systems beyond the dopaminergic component is far from elucidated, and although speculative, the neuroanatomical correlation and relevance of these signaling alterations are an important topic for further investigation, with possible highly-sought-after therapeutic implications for the clinical practice.

## Author contributions

PF, PL, PH, MBal, MBar, and TK participated in designing the project and defining the aims and hypotheses. PF, RŠ, MBal, and MBar were responsible for patient recruitment and neurological examinations. PL and PH performed neuropsychological testing. PF performed the data analysis and wrote the manuscript draft. All co-authors provided their comments to the manuscript draft.

### Conflict of interest statement

The authors declare that the research was conducted in the absence of any commercial or financial relationships that could be construed as a potential conflict of interest.

## Abbreviations

MRI: magnetic resonance imaging
fMRI: functional magnetic resonance imaging
BOLD: blood-oxygen-level dependent
SPECT: Single-photon emission computed tomography
PD: Parkinson's disease
ICD: impulse control disorder
GNG: Go/No Go task
DD: Delay Discounting task.
